# Combined Use of GDF-15 and NT-Pro BNP for Outcome Prediction in Patients with Acute Heart Failure

**DOI:** 10.3390/jcm13195936

**Published:** 2024-10-05

**Authors:** Joanna Płonka, Anna Klus, Natalia Wężyk, Klaudia Dąbrowska, Lidia Rzepiela, Ewa Gawrylak-Dryja, Krzysztof Nalewajko, Piotr Feusette, Marek Gierlotka

**Affiliations:** 1 Department of Cardiology, University Hospital, Institute of Medical Sciences, University of Opole, 45-401 Opole, Poland; natalia_wezyk@o2.pl (N.W.); klaudia.da@gmail.com (K.D.); lrzepiela@wp.pl (L.R.); krzysztof.nalewajko@uni.opole.pl (K.N.); feusette@wp.pl (P.F.); marek.gierlotka@gmail.com (M.G.); 2 Department of Clinical Biochemistry and Laboratory Diagnostics, Institute of Medical Sciences, University of Opole, 45-401 Opole, Poland; klusanna@interia.pl (A.K.), ewa.gawrylak-dryja@usk.opole.pl (E.G.-D.)

**Keywords:** acute heart failure, biomarkers, serial measurement, risk stratification

## Abstract

**Background:** Acute heart failure (AHF) is characterized by a complex pathophysiology. **Aims:** This study aimed to evaluate the usefulness of combined serial measurements of N-terminal pro-B-type natriuretic peptide (NT-pro BNP) and growth differentiation factor 15 (GDF-15) for predicting long-term outcomes in patients with AHF. **Methods:** This study included 104 consecutive patients hospitalized due to AHF. The mean (SD) age was 65 (±15) years. Blood samples were collected on admission, at discharge, and at a 30-day follow-up visit. The primary composite endpoint was all-cause mortality or rehospitalization due to heart failure (HF) at 1-year follow-up. **Results:** During follow-up, the primary endpoint occurred in 31 persons. In the ROC analysis, the optimal cut-off values of GDF-15 for predicting the outcome were 5115.5 pg/mL on admission, 4145 pg/mL at discharge, and 4218.5 pg/mL at the 30-day visit. For NT-pro BNP, the optimal cut-off reached 6011 ng/L, 1250 ng/L, and 1456.5 ng/L, respectively. Patients with both GDF-15 and NT-pro BNP levels above the cut-off value had a higher risk of the primary composite endpoint than patients with only one or none of the biomarkers elevated at three time points. At the 30-day visit, the model combining NT-pro BNP and GDF-15 showed the highest predictive value for the primary composite endpoint (area under the curve, 0.75). **Conclusions:** Combined serial measurements of NT-pro BNP and GDF-15 outperform single measurements in outcome prediction at 1-year follow-up in patients with AHF. The repetitive combined model may serve as a useful risk assessment tool and facilitate decision-making during long-term observation.

## 1. Introduction

Acute heart failure (AHF) is a complex condition with different phenotypes [1]. It is the leading cause of hospitalization in patients above 65 years of age and described as a global pandemic, constituting an increasing financial burden for healthcare systems worldwide [2,3].

Natriuretic peptides, including B-type natriuretic peptide (BNP) and N-terminal pro-B-type natriuretic peptide (NT-pro BNP), are standard laboratory biomarkers for the diagnosis of HF and risk stratification of patients. However, as the pathophysiology of AHF is heterogeneous, new biomarkers or prognostic models are still needed to improve risk stratification, assess the clinical course of the disease, and facilitate clinical decision-making [4]. Data from recent meta-analyses and systemic literature reviews indicate GDF-15 as an emerging and independent predictor of mortality and adverse outcomes in HF patients [5].

Growth differentiation factor 15 (GDF-15) is a stress response protein with pleiotropic effects and a member of the transforming growth factor-β superfamily. This novel prognostic marker for patients with AHF was reported to be noninferior to NT-pro BNP [6]. GDF-15 is expressed at low levels in healthy humans, except in pregnant women, who have high GDF-15 expression in the placenta [7,8]. Its elevated values are associated with a higher risk of cardiovascular disease, chronic kidney disease, and cancer. Some authors reported GDF-15 as a marker of biological age, with low serum GDF-15 levels indicating general health and longevity [9]. Injury, ischemia, and stimulation by proinflammatory cytokines result in higher GDF-15 expression in macrophages, while ischemia–reperfusion injury, mechanical stretch, and pressure overload lead to GDF-15 secretion by cardiomyocytes [10,11]. Moreover, it serves as a marker of death and various complications in numerous cardiovascular diseases [11]. A substantial body of evidence supports the prognostic utility of GDF-15 in patients with acute coronary syndrome and HF, as well as its usefulness for predicting bleeding complications in acute pulmonary embolism and atrial fibrillation in patients on anticoagulation therapy [12,13,14,15,16,17,18,19,20]. While GDF-15 can be produced by the heart, extracardiac tissues remain the primary source of this biomarker. Following secretion, GDF-15 acts as an inflammatory interaction regulator in an autocrine, paracrine, and endocrine manner [21]. Recent scientific studies in animal models revealed that blocking GDF-15 reduces severe HF and stops cardiac cachexia. Elevated GDF-15 could probably act as a therapeutic target among HF patients [22].

The aim of this study was to assess the changes in serum levels of NT-pro BNP and GDF-15 during an episode of AHF and to determine the utility of combined serial measurements of GDF-15 and NT-pro BNP for risk stratification of AHF patients.

## 2. Materials and Methods

### 2.1. Study Design

This was a single-center study based on the Open Prospective Acute Heart Failure (OP-AHF) Registry. AHF was recognized by the typical signs and symptoms (breathlessness, pulmonary crackles, peripheral edema, ankle swelling, fatigue, and elevated jugular venous pressure). Each patient underwent a cardiovascular examination including clinical history, basis of physical examination, 12-lead electrocardiogram, laboratory tests, chest X-ray, lung ultrasound, and transthoracic echocardiography performed within 24 h of admission [14]. The inclusion criteria were age over 18 years and hospitalization due to AHF requiring the use of at least one of the following: intravenous diuretics, catecholamines, or mechanical circulatory support. The exclusion criteria were a lack of patient consent, active malignancy, autoimmune disease, and psychiatric disorders. The final sample included 104 consecutive patients admitted to the intensive cardiac care unit between June 2019 and January 2021. All patients were managed in accordance with the relevant guidelines for the diagnosis and treatment of AHF, and the treatment was initiated at the discretion of the attending cardiologist [1]. The clinical outcome was a composite endpoint of one-year all-cause mortality or rehospitalization due to HF.

### 2.2. Biochemical Analysis

Blood samples were collected within 24 h of admission, at discharge, and during an ambulatory visit at 30 days after discharge. Each sample was immediately centrifuged at 4000 RPM for 15 min to obtain a plasma supernatant and then frozen at a temperature of −80 °C until analysis. Plasma GDF-15 levels were measured using an automated sandwich electrochemiluminescence immunoassay with a reference range of 400 to 20,000 ng/L (Roche Diagnostics GmbH, Mannheim, Germany). Serum NT-pro BNP levels were measured quantitatively using an automated sandwich electrochemiluminescence immunoassay (Roche Diagnostics GmbH). An NT-pro BNP level above 300 pg/mL was considered elevated.

### 2.3. Statistical Analysis

Data were presented as a mean with standard deviation and a median with interquartile range (IQR) for quantitative variables and as counts and percentages for categorical variables. The normality of distribution was verified using the Shapiro–Wilk test. Clinical parameters were compared between 2 groups using the Mann–Whitney test for variables with non-normal distribution and the Student *t*-test for those with normal distribution. The chi-square test with Yates correction was used for categorical variables. The results on graphs are expressed as box plots. The pairwise test was used to compare 2 parameters within a single group. Cumulative survival data were assessed using the Kaplan–Meier method. The optimal cut-off values of GDF-15 and NT-pro BNP for predicting the primary endpoint were calculated using a receiver operating characteristics (ROC) curve analysis. The associations of GDF-15 and NT-pro BNP levels with the study endpoints were established using univariable and multivariable Cox proportional hazard analyses. The number of elevated biomarkers was included as a separate categorical variable to highlight information about the diverse pathophysiology. A *p*-value of less than 0.05 was considered significant. Statistical analyses were performed using the R software v.4.2.2 (The R Foundation for Statistical Computing, Vienna, Austria).

### 2.4. Follow-Up

Follow-up visits were scheduled for all patients at 30 days after discharge from hospital. In addition to blood sample collection, a cardiovascular work-up followed by a 12-lead electrocardiogram were performed. At the follow-up visit 1 year after hospital discharge, we obtained data from patients, families, the general practitioner, or using hospital records. In the case of death, information was acquired by phone from relatives and death certificates.

## 3. Results

### 3.1. Study Population

A total of 104 patients were included in the study. During the 1-year follow-up, the primary endpoint of all-cause mortality or rehospitalization due to HF occurred in 31 patients (30%) (Figure S1). The mean (SD) age of patients was 65 (±15) years, and 74% of patients were male. New-onset AHF was reported in 49% of the population. A history of coronary artery disease was reported in 44% of the population. Patients, at admission time, presented a significant HF (68% NYHA class IV) with a median left ventricular ejection fraction of 30% (IQR, 20–38%). A non-ischemic etiology of AHF occurred in 55%. Patients in critical condition were treated with catecholamines (15%) or mechanical circulatory support (6%). Patients who met the primary endpoint were older (*p* = 0.04) and had more comorbidities. They also had higher levels of established biomarkers of myocardial overload and injury, NT-pro BNP, and hs-TNT, as well as a significantly increased values of creatinine and bilirubine. The clinical characteristics of the study population are presented in Table 1.

### 3.2. Association of GDF-15 and NT-Pro BNP Levels with Study Endpoints

**Figure 1:**
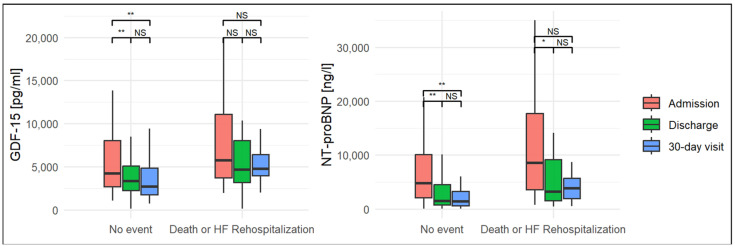
Changes in GDF-15 and NT-pro BNP levels divided by outcome in the one-year follow-up period. * *p* = 0.01, ** *p* < 0.01, NS—not significant.

In the whole cohort, GDF-15 and NT-pro BNP levels on admission were strongly elevated. The median values of biomarkers on admission were as follows: GDF-15, 4563 pg/mL (2951–9072 pg/mL); NT-pro BNP, 5676 ng/L (2932–3235 ng/L). The biomarker levels decreased from admission to discharge: GDF-15 3633 pg/mL (2444–6218 pg/mL); NT-pro BNP 1684 ng/L (918–7410 ng/L). At 30-day follow-up, they were as follows: GDF-15 2953 pg/mL (1973–5363 pg/mL), NT-pro BNP 1902 ng/L (746–4102 ng/L). Noticeable differences in GDF-15 and NT-pro BNP levels occurred in the study group during observation. The NT-pro BNP levels were significantly higher at three time points in the patients who subsequently died or required HF rehospitalization compared to the event-free group. Patients who met the primary endpoint were characterized by significantly higher levels of GDF-15. Only the GDF-15 values at discharge were not notably different, as shown in Table 1. The kinetics of GDF-15 and NT-pro BNP in the group free from events were characterized by a decreasing pattern. In the subgroup with death or HF rehospitalization, the kinetics of both biomarkers were flat. The kinetics of both biomarkers divided by the outcome are presented in Figure 1.

In the ROC analysis, the optimal cut-off values of GDF-15 for predicting the outcome were 5115.5 pg/mL on admission, 4145 pg/mL at discharge, and 4218.5 pg/mL at the 30-day visit. For NT-pro BNP, the optimal cut-off reached 6011 ng/L, 1250 ng/L, and 1456.5 ng/L, respectively (Figure S2). Based on the optimal cut-off levels, patients were classified into four subgroups:

(1) GDF-15 and NT-pro BNP levels below the cut-off value;
(2) Only GDF-15 levels above the cut-off value;
(3) Only NT-pro BNP levels above the cut-off value;
(4) Both GDF-15 and NT-pro BNP levels above the cut-off value.

**Figure 2:**
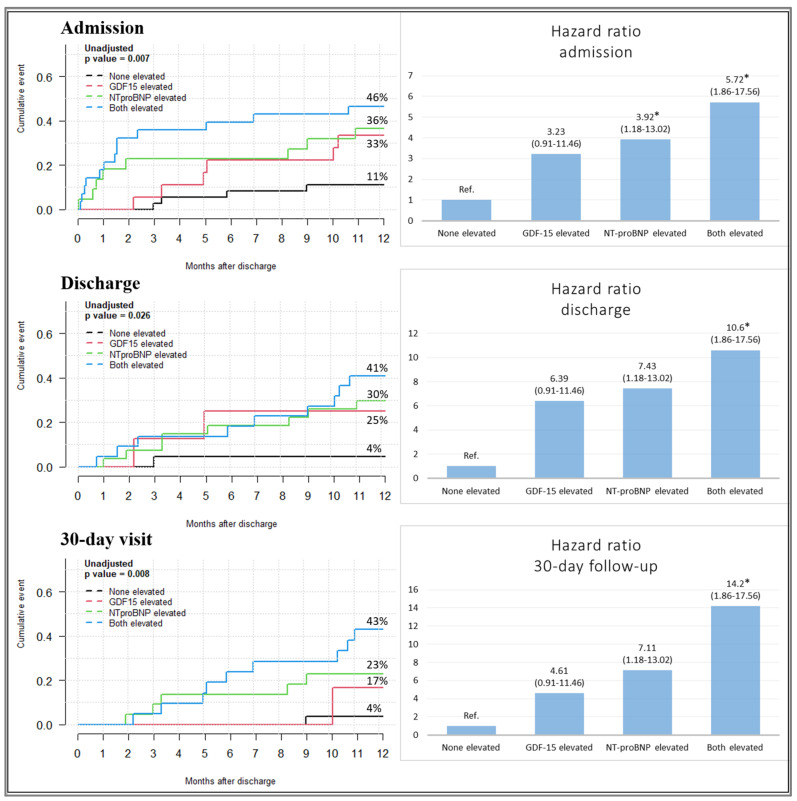
The Kaplan–Meier curves and relationship between the number of elevated biomarkers NT-pro BNP and GDF-15 and the 1-year primary composite endpoint (univariate Cox analysis: * *p* < 0.05 vs. patients with no biomarker being elevated) at admission, discharge, and 30-day visit.

The Kaplan–Meier analysis on admission, at discharge, and at the 30-day visit was used for predicting the risk of the primary composite endpoint during the 1-year follow-up. Patients with both NT-pro BNP and GDF-15 levels above the cut-off values had higher rates of all-cause mortality or HF rehospitalization than those with only one or none of the biomarkers elevated (46%, 41%, and 43%, respectively), independently of the time point, as shown in Figure 2. The graded relationship between the number of elevated biomarkers at admission, discharge, 30-day visit, and 1-year primary composite endpoint revealed nearly 6 times, 10 times, and 14 times higher risks of 1-year all-cause mortality or HF rehospitalization compared with patients without elevated values of biomarkers. These findings demonstrate the potential of the composite diagnosis.

Based on the clinical characteristics, significant variables were selected and included in the Cox regression hazard model. The risk of the composite endpoint for the patients with both biomarkers, GDF-15 and NT-pro BNP, above the cut-off value was twice as high at 30-day follow-up than on admission and at discharge, as shown in Table 2.

The area under the ROC curve (AUC) for GDF-15 and NT-pro BNP for predicting all-cause mortality or HF rehospitalization at three time points are presented in Figure S2. However, there were no differences in the AUC between the biomarkers at the admission time, discharge, and 30-day visit, while the model combining both biomarkers had the best predictive value at 30 days (AUC, 0.75 [95% CI 0.63–0.86], *p* = 0.001). When comparing the combination of both biomarkers with GDF-15 or NT-pro BNP alone, there were also no significant differences at any of the time points tested (Figure S2).

## 4. Discussion

The key finding of our study is that serial measurements of NT-pro BNP and GDF-15 dynamically predict all-cause mortality and HF rehospitalization in patients with AHF during the 1-year follow-up period. The model combining both biomarkers, GDF-15 and NT-pro BNP, provides a useful tool for risk stratification of patients with AHF.

The natriuretic peptides BNP and NT-pro BNP are secreted in response to neurohormonal activation and constitute gold-standard biomarkers in HF. However, HF has been shown to have complex pathophysiology, and several biomarkers reflecting different pathophysiological have been identified [23]. GDF-15 is involved in inflammatory processes and myocardial remodeling, which constitutes a distinct pathophysiological pathway in HF.

Analysis of the COMBINE-AF cohort showed that NT-pro BNP, hs TnT, and GDF-15 were significantly associated with a high risk HF events among patients with atrial fibrillation (AF). The use of these biomarkers for selecting low- and high-risk AHF patients seems possible in the near future [24].

In the BioPredict study assessing candidate biomarkers in patients with chronic HF, only 4 of the 11, including GDF-15, were independent predictors of the study endpoints [25]. Moreover, the study showed that none of the biomarkers significantly increased the predictive value of the study endpoints. However, among the study limitations, the authors noted that the biomarkers were determined at a single time point, and that longer monitoring of these parameters would be needed to better assess the usefulness of the prediction model based on multiple biomarkers.

There is no doubt that AHF is different from chronic HF. AHF is a dynamic process, and many imaging, as well as laboratory parameters, including biomarkers, change during treatment. These biomarker’s kinetics can facilitate risk stratification and therapeutic decision-making [1,26]. Chinese investigators measured GDF-15 and NT-pro BNP levels on admission in a population of 260 patients with AHF [6]. GDF-15 was found to be noninferior to NT-pro BNP in predicting death at 1 year. In recent years, the results of studies assessing multiple biomarkers at multiple time points in patients with AHF have been published, providing significant insights into the prediction of death and rehospitalization [27,28].

In line with the TRIUMPH study, in our study, we reported high levels of serial GDF-15 measurements and, importantly, weak kinetics without significant changes in biomarker levels in patients who experienced death or HF rehospitalization at the 1-year follow-up. The NT-pro BNP values were also significantly elevated in this subgroup and dropped between admission and discharge, but they remained at a similar level between discharge and the 30-day follow-up. Small changes in the levels of GDF-15 and NT-pro BNP indicate high-risk patients who do not respond to the standard medical treatment for HF, as shown in Figure 1.

The Kaplan–Meier survival curves, including the number of elevated biomarkers, showed that patients with both GDF-15 and NT-pro BNP levels above the cut-off point had a significantly higher risk of death or HF rehospitalization at each of the three time points than patients with only one or no elevated biomarkers. This simple model can be used for the early identification of patients in the phenotypically diverse AHF population who are at highest risk of a poor prognosis and require close monitoring, intensive diagnostic procedures, and special therapeutic management (Figure 2).

The AUC values determined separately for NT-pro BNP and GDF-15 at the three time points were similar, while the best prognostic value was obtained for the combination of NT-pro BNP and GDF-15 at the 30-day follow-up. Serial measurements of biomarkers show that the risk of death or HF rehospitalization is variable and can be modified. High biomarker levels that persist despite treatment are associated with poor prognosis; therefore, similar to the authors of the RELAX- HF study [27], we believe that measuring biomarkers in the post-baseline period has a better predictive value in long-term prognostication. In our study, the combination of NT-pro BNP and GDF-15 had a better predictive value than a single measurement of individual biomarkers at each of the three time points. In the multivariable analysis, the model combining both NT-pro BNP and GDF-15 showed a 2-fold higher risk of the outcome occurring at the 30-day follow-up compared with admission and discharge, as shown in Table 2. Our observations are consistent with the findings of other investigators [29], who found that biomarkers representing different pathophysiological pathways can provide additional information and offer better risk stratification in patients with AHF than a single biomarker. The use of serial measurements of both biomarkers can improve the identification subset of high-risk patients who require close monitoring and are candidates to complex procedures. The early identification of AHF patients improves their prognosis.

Our study has several limitations. First, it was a single-center analysis including a small sample of patients. Second, the levels of biomarkers can vary from day to day and may be affected by fasting. A large multicenter study is needed to confirm our results.

## 5. Conclusions

Serial measurements of multiple biomarkers among acute heart failure patients are promising and provide a better prognostic value than the measurement of a single marker at a single time point. A complex model, including both NT-pro BNP and GDF-15, may play a supplemental role to support the treatment based only on BNP and better predict all-cause mortality and HF rehospitalization during 1-year follow-up.

## Figures and Tables

**Table 1:** Clinical characteristics of the study population.

Variable	Event-Free Patients (*n* = 73)	Death or HF Rehospitalization (*n* = 31)	*p*-Value
Demographic data			
Age, years, mean (SD)	63 (±17)	71 (±7)	0.04
Male, *n* (%)	50 (69)	27 (81)	0.05
New onset AHF, n (%)	40 (55)	11 (36)	0.07
BMI, kg/m^2^, mean (SD)	31 (±6)	27 (±5)	0.02
Medical history			
Hypertension, *n* (%)	48 (66)	24 (77)	0.24
CAD, *n* (%)	24 (33)	21 (70)	0.11
Diabetes, *n* (%)	22 (30)	17 (55)	0.02
CKD, *n* (%)	15 (21)	14 (45)	0.01
Atrial fibrillation, *n* (%)	32 (44)	16 (52)	0.47
ICD/CRTD/CRT, *n* (%)	12 (16)	12 (39)	0.35
Etiology of AHF			
Ischemic, *n* (%)	25 (34)	22 (71)	<0.001
Valve disease, *n* (%)	25 (34)	17 (55)	0.05
Inflammatory, *n* (%)	14 (19)	1 (3)	0.15
DCM, *n* (%)	6 (8)	2 (7)	0.76
Arrhythmic, *n* (%)	9 (12)	5 (16)	0.60
Hospitalization data			
HR, bpm, mean (SD)	110 (±28)	94 (±33)	0.03
SBP, mmHg, mean (SD)	135 (±25)	123 (±25)	0.04
NYHA class IV, *n* (%)	47 (64)	24 (77)	0.25
Hospital stay, days median (IQR)	15 (12–23)	15 (8–28)	0.35
Norepinephrine, *n* (%)	8 (11)	8 (26)	0.06
Dobutamine, *n* (%)	6 (8)	7 623)	0.04
Diuretics iv, *n* (%)	68 (93)	30 (97)	0.47
MCS–IABP, *n* (%)	4 (6)	2 (7)	0.85
β-blocker, *n* (%)	67 (92)	24 (77)	0.04
ARB, *n* (%)	7 (10)	2 (7)	0.60
ACEI, *n* (%)	51 (70)	15 (48)	0.04
MRA, *n* (%)	54 (73)	9 (29)	0.05
ARNI, *n* (%)	8 (11)	8 (26)	0.06
SGLT2 inhibitor, *n* (%)	6 (8)	1 (3)	0.35
Laboratory and echocardiographic parameters		
GDF-15, pg/mL, median (IQR)		
admission	4216 (2717–8036)	5735 (3704–11,103)	0.03
discharge	3351 (2267–5108)	4667 (3195–8056)	0.10
30-day visit	2718 (1741–4854)	4736 (3988–6438)	0.01
NT-pro BNP, ng/L, median (IQR)		
admission	4805 (2124–10,108)	8522 (3628–17,762)	0.02
discharge	1452 (801–4538)	3222 (1583–9193)	0.03
30-day visit	1412 (648–3249)	3843 (1920–5744)	0.01
hsTnT, ng/L, median (IQR) admission	41 (27–133)	80 (31–533)	0.09
Creatinine, mg/dL, median (IQR) admission	1.2 (0.97–1.36)	1.3 (1.1–2.2)	0.04
Bilirubin, mg/dL, median (IQR) admission	0.7 (0.53–1.42)	1.5 (0.72–1.79)	0.05
Hemoglobin, g/dL, median (IQR) admission	14 (11–15)	12 (10.7–13.6)	0.02
LVEF, %, mean (SD)	32 (±15)	28 (±10)	0.31
LVEDd, mm, mean (SD) admission	60 (±9)	61 (±10)	0.45

Abbreviations: ACEI, angiotensin-converting enzyme inhibitor; ADHF, acute decompensated heart failure; AHF, acute heart failure; ARB, angiotensin receptor blocker; ARNI, angiotensin receptor-neprilysin inhibitor; BMI, body mass index; CAD, coronary artery disease; CKD, chronic kidney disease; CRT, cardiac resynchronization therapy; CRTD, cardiac resynchronization therapy with defibrillator function; DCM, dilated cardiomyopathy; GDF-15, growth differentiation factor 15; HR, heart rate; hsTn, high-sensitivity troponin T; IABP, intra-aortic balloon pump; ICD, implantable cardioverter defibrillator; IV, intravenous; LVEF, left ventricular ejection fraction; LVEDd, left ventricular end-systolic dimension; MCS, mechanical circulatory support; MRA, mineralocorticoid receptor antagonist; NT-pro BNP, N-terminal pro-B-type natriuretic peptide; NYHA, New York Heart Association; SBP, systolic blood pressure; SGLT2, sodium-glucose cotransporter-2.

**Table 2:** Predictors of 1-year all-cause mortality or HR rehospitalization in patients admitted due to acute heart failure.

Univariable Model		
Variable	HR (95% CI)	*p*-value
NT-pro BNP and GDF-15 elevated admission	5.56 (1.82–17.18)	0.01
NT-pro BNP and GDF-15 elevated discharge	10.81 (1.37–85.41)	0.02
NT-pro BNP and GDF-15 elevated 30-day visit	14.57 (1.84–115.23)	0.01
Age	1.03 (1.01–1.06)	0.02
Ischemic etiology	3.63 (1.67–7.89)	0.001
Diabetes	2.31 (1.14–4.69)	0.02
Multivariable model		
Variable	HR (95% CI)	*p*-value
Admission		
NT-pro BNP and GDF-15 elevated	4.38 (1.24–15.5)	0.02
Age	1.03 (0.98–1.08)	0.25
Ischemic etiology	2.35 (0.69–8.02)	0.17
Diabetes	0.88 (0.24–3.17)	0.88
Discharge		
NT-pro BNP and GDF-15 elevated	5.62 (0.55–50.10)	0.15
Age	1.05 (0.97–1.12)	0.24
Ischemic etiology	0.43 (0.11–1.72)	0.23
Diabetes	4.86 (0.77–30.67)	0.09
30-day visit		
NT-pro BNP and GDF-15 elevated	10.15 (0.91–112.72)	0.06
Age	1.00 (0.93–1.09)	0.89
Ischemic etiology	1.42 (0.27–7.45)	0.62
Diabetes	1.34 (0.28–6.46)	0.71

## Data Availability

All relevant data are contained within the manuscript.

## Supplementary Materials

The following supporting information can be downloaded at https://www.mdpi.com/article/10.3390/jcm13195936/s1: Figure S1: Flowchart of the 1-year follow-up in patients with acute heart failure; Figure S2: Pairwise comparison of the area under the curves of GDF-15 and NT-pro BNP in predicting 1-year all-cause mortality or HF rehospitalization.
