# Supplementary figures and images for: Combined Use of GDF-15 and NT-Pro BNP for Outcome Prediction in Patients with Acute Heart Failure
Source: J Clin Med. 2024 Oct 5;13(19):5936. doi: 10.3390/jcm13195936 (PMC11477740; doi:10.3390/jcm13195936)

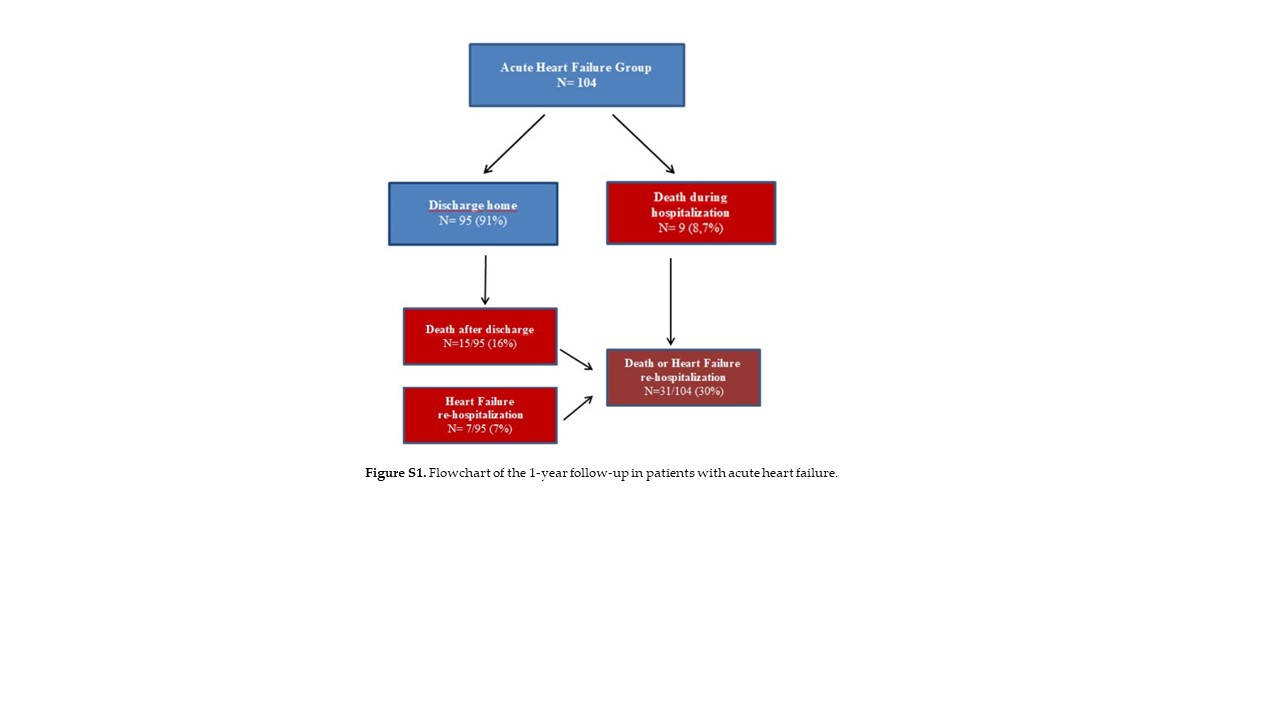

Supplement: Supplementary file 1 [file jcm-13-05936-s001.zip › Figure S1 GDF-15+ NT- pro BNP (11.09.2024).jpg]

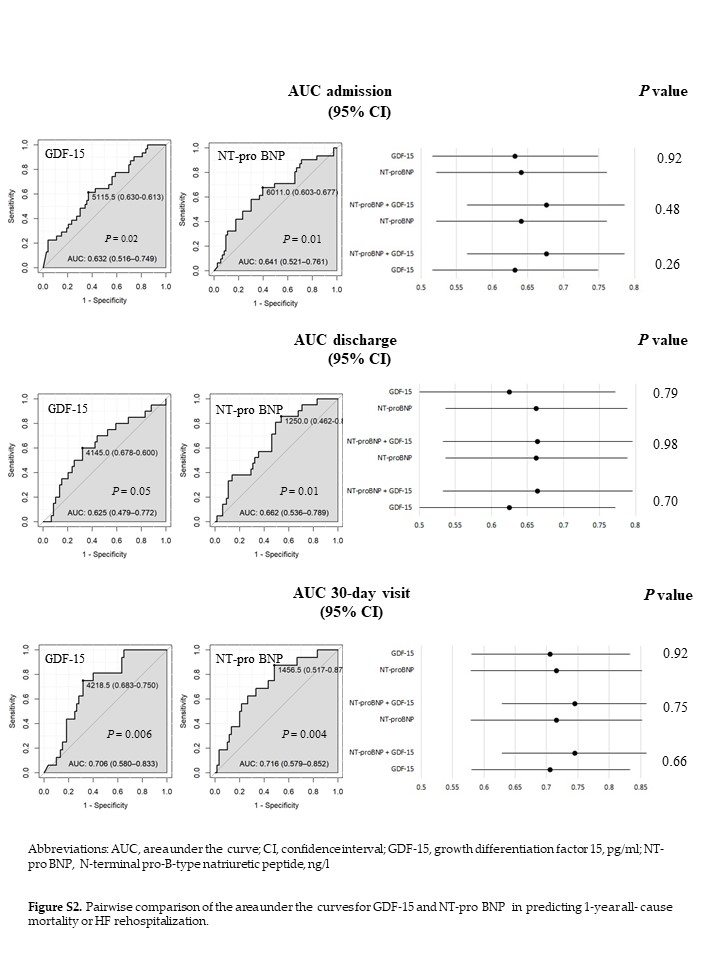

Supplement: Supplementary file 1 [file jcm-13-05936-s001.zip › Figure S2 GDF+ BNP ( ROC) 11.09.2024.jpg]
